# Negative association of steroids with immunotherapy efficacy in a multi-tumor cohort: time and dose-dependent

**DOI:** 10.1007/s00262-024-03772-9

**Published:** 2024-08-02

**Authors:** Víctor Albarrán, Patricia Guerrero, Coral García de Quevedo, Carlos González, Jesús Chamorro, Diana Isabel Rosero, Jaime Moreno, Juan Carlos Calvo, Patricia Pérez de Aguado, Víctor Alía, Pilar Sotoca, Ana María Barrill, María San Román, Pablo Álvarez-Ballesteros, Juan José Serrano, Ainara Soria, María Eugenia Olmedo, Cristina Saavedra, Alfonso Cortés, Ana Gómez, Yolanda Lage, Álvaro Ruiz, María Reyes Ferreiro, Federico Longo, Pilar Garrido, Pablo Gajate

**Affiliations:** Department of Medical Oncology, Ramon y Cajal University Hospital (Madrid), Instituto Ramón y Cajal de Investigación Sanitaria (IRYCIS), Madrid, Spain

**Keywords:** Steroids, Immunotherapy, Immune checkpoint inhibitors, Dose, Neutrophil-to-lymphocyte ratio, Solid tumors

## Abstract

Previous studies have suggested a negative impact of steroids on the efficacy of immune checkpoint inhibitors (ICI), but how this effect is modulated by the dosage and time of administration is yet to be clarified. We have performed a retrospective analysis of 475 patients with advanced solid tumors treated with ICI as monotherapy from 2015 to 2022. Data regarding immune-related adverse events (irAEs) and clinical outcomes were collected. For each patient, the daily steroid dose (in mg/kg of prednisone) was registered until disease progression or death. The impact of cumulative doses on response rates and survival outcomes was analyzed within different periods. The objective response rate (ORR) was significantly lower among patients exposed to steroids within 30 days before the first cycle of ICI (C1) (20.3% vs. 36.7%, *p* < 0.01) and within the first 90 days of treatment (25.7% vs. 37.7%, *p* = 0.01). This negative association was confirmed by multivariable analysis. Higher mean steroid doses were observed among non-responders, and cumulative doses were inversely correlated with the disease control rate (DCR) around ICI initiation. Remarkably, poorer outcomes were observed even in patients belonging to the lowest dose quartile compared to the steroid-naïve population. The exposure to steroids after 6 months of ICI was not associated with worse survival outcomes. Our results suggest that the potential impact of steroids on ICI efficacy may be time-dependent, prevailing around ICI initiation, and dose-dependent, with modulation of neutrophil-to-lymphocyte ratio as a possible underlying mechanism.

## Introduction

Immune checkpoint inhibitors (ICI) have changed the therapeutic landscape of many solid tumors. However, huge variability in immunogenicity and responsiveness to treatment has been observed among different patients. This is a highly complex phenomenon influenced by multiple biomarkers, including tumor mutational burden (TMB) [1], PD-L1 expression [2], quantity and characteristics of infiltrating T lymphocytes [3], B cells phenotype [4] and the interaction between the immune system and the TME [5], among others. The presence of immunosuppressive cells in the TME, such as myeloid-derived suppressor cells (MDSCs [6]), tumor-associated macrophages (TAMs [7]) and regulatory T cells (T-reg [8]), strongly interferes with the efficacy of the anti-tumor response, leading to immune-resistant ‘cold’ tumors and promoting tumor progression.

Steroids have anti-inflammatory and immunosuppressive effects, which imply both innate and adaptive immunity. Glucocorticoid receptor (GR) activation alters the transcription of numerous genes encoding pro-inflammatory cytokines (NF-kB, AP-1) and genes related to T-cell survival, trafficking, and cytotoxic activity [9]. Steroids have been proven to reduce immune cell infiltration [10], facilitate MDSCs expansion [11], promote T-reg proliferation, and enhance the production of T-reg-activating cytokines (such as TGF-β) [12]. Increased GR signaling has been correlated with CD8 + TILs dysfunction and upregulation of several immune checkpoints [13].

Steroids are frequently prescribed for patients with advanced solid tumors owing to a wide range of clinical scenarios, including symptomatic brain metastasis, chemotherapy-induced emesis, cancer-related complications, and additional chronic comorbidities. In addition, except for endocrine toxicity, steroids are the mainstay of management for grade ≥ 2 immune-related adverse events (irAEs), usually starting at 0.5–2 mg/kg/day of prednisone until clinical improvement, and subsequent slow tapering over 4–6 weeks [14]. Given their immunosuppressive properties, there is growing concern regarding their detrimental effects on the clinical outcomes of immunotherapy.

Although the negative impact of steroid exposure has been shown in several studies, how the dosage and time of administration modulate this effect is yet to be clarified. A better understanding of these aspects would be of great value in optimizing steroid use in oncological patients. In this real-world data (RWD) study, we aimed to collect detailed information on the dose and timing of steroid administration in a multi-tumor cohort of patients treated with ICI and to evaluate their impact on clinical outcomes.

## Methods

### Study design and population

This is a retrospective single-center study performed after approval from the Clinical Investigation Ethical Committee (CEIC) of our institution (Ramon y Cajal University Hospital, Madrid), including adult patients (> 18 years old) with metastatic and/or unresectable solid tumors who received systemic treatment with ICI between April 2015 and October 2022. Patients who received ICI in the context of a clinical trial, as well as those treated with combinations of ICI with chemotherapy, tyrosine-kinase inhibitors, or any other anticancer agents, were excluded from the analysis. In accordance with the CEIC, verbal informed consent was obtained from all patients. Clinical data were extracted from pharmacy databases and electronic institutional medical files.

For the selected patients, we obtained data regarding their demographics, previous medical history (including autoimmune disease or human immunodeficiency virus [HIV] infection), oncologic history (tumor histology, date of diagnosis, cancer stage according to the American Joint Committee on Cancer [AJCC] staging system, disease burden, previous treatments), performance status (PS) according to the Eastern Cooperative Oncology Group (ECOG) score [15], treatment with ICI (including type of ICI, treatment duration, and immune-related toxicity) and laboratory data, including neutrophil-to-lymphocyte (N/L) ratio. We defined the date of progression as either that of radiological (according to RECIST criteria V.1.1) or clinical evidence of progressive disease. The objective response rate (ORR) was defined based on the best objective response. The disease control rate (DCR) was defined as objective response or stable disease (SD) at least on the first radiological evaluation (generally 10–12 weeks after treatment initiation).

For each patient, data regarding steroid administration were collected from 30 days before the first cycle of ICI (C1) until disease progression or death, including the type of steroids, clinical indication, and daily dose in mg/kg of prednisone (using the following dose conversion: 1 mg of dexamethasone = 6.67 mg of prednisone; 1 mg of metilprednisolone = 1.25 mg of prednisone; 1 mg of hydrocortisone = 0.25 mg of prednisone; 1 mg of deflazacort = 0.67 mg of prednisone). Patients without detailed information regarding steroid dosage were excluded from the study. For each patient, we calculated the total steroid dose within different periods of time: 30 days before C1 (-30D), 30 days after C1 (D1-30), 90 days after C1 (D1-90), and after 6 months from C1 (> 6 m) in those without disease progression (irrespective of previous steroids exposure within the first 6 months).

### Statistical analysis

Statistical analysis was performed using the STATA software. The distribution of qualitative variables was summarized using frequencies and percentages, and the associations between them were evaluated using Fisher’s exact or chi-square tests. The distribution of each continuous variable was summarized using mean and standard deviation. Independent sample T-test was used to analyze quantitative variables between different groups of patients, assuming a normal distribution. Survival outcomes were estimated using Kaplan–Meier curves, and a Cox proportional hazard regression model was used for multivariable analysis. Statistical significance was set at *P* values < 0.05. All statistical evaluations were two sided.

## Results

### General characteristics

A total of 475 patients with advanced solid tumors were included in the analysis (66.7% male), with a median age of 67.5 years. The tumor subtype distribution was as follows: 161 patients with non-small cell lung cancer (NSCLC) (33.9%), 82 with urothelial cancer (17.3%), 65 with renal cancer (13.7%), 53 with melanoma (11.2%), 52 with head and neck squamous tumors (11.0%), 15 with gastrointestinal tumors (3.2%), 11 with gynecological tumors (2.3%), 6 with anal canal carcinoma (1.3%), 5 with malignant mesothelioma (1.1%), 5 with skin epidermoid tumors (1.1%), and 18 with other tumors (3.8%). Thirty-four patients (7.2%) with a previous diagnosis of autoimmune disease and thirteen patients (2.7%) with a controlled HIV infection were included.

Regarding the type of ICI, 306 patients (64.4%) were treated with anti-PD-1 monotherapy (161 pembrolizumab, 136 nivolumab, 8 cemiplimab, and 1 spartalizumab), 97 patients (20.4%) received anti-PD-L1 monotherapy (81 atezolizumab, 14 avelumab, and 2 durvalumab), 63 patients (13.3%) were treated with combined anti-PD-1 + anti-CTLA-4 (nivolumab plus ipilimumab), and 9 patients (1.9%) received anti-CTLA-4 (ipilimumab) as monotherapy. A total of 200 patients (42.1%) received ICI as the 1st line of treatment, 205 patients (43.2%) as 2nd line, and 70 patients (14.7%) in 3rd or further lines. The baseline ECOG PS was 0 in 147 patients (31.0%), 1 in 254 patients (53.6%), and ≥ 2 in 73 patients (15.4%). Immune-related adverse events (irAEs) were diagnosed in 157 patients (33.1%), with grade 3–4 events in 55 (11.6%) and one toxic death (0.2%) (pembrolizumab-related Stevens-Johnson syndrome). The most frequent irAEs were colitis (7.6%), hypothyroidism (7.0%), cutaneous toxicity (6.3%), nephritis (4.4%) and hepatitis (3.4%). The baseline neutrophil-to-lymphocyte (N/L) ratio was > 4 in 197 patients (42.7%).

Steroid use was documented in 229 patients (48.2%). 82 patients received steroids within 30 days prior to C1 of ICI (17.2%), mainly due to cancer-related symptoms (71.8%). Dexamethasone was the most frequently administered drug (73.1%). After ICI initiation, 105 patients (22.1%) received steroids for immune-related toxicity (5.7% in D1-30, 10.7% in D1-90, and 12% in > 6 months). 159 patients (33.5%) received steroids for other reasons (20.6% in D1-30, 25.1% in D1-90, and 8% in > 6 months). In total, 155 patients received steroids within 90 days after C1 (32.6%). Among the 182 patients (38.3%) who remained with responsive or stable disease at 6 months, 79 (43.4%) received steroids after 6 months from C1.

The baseline characteristics of the patients, divided into groups based on steroid use during different periods of time, are summarized in Table 1.

**Table 1:** Baseline characteristics, patients were grouped according to steroid use over different periods of time. ST: steroids; ICI: immune checkpoint inhibitor; -30D: 30 days before the first cycle of ICI (C1); D1-90: 90 days after C1; > 6 m: after 6 months from C1 (in those without disease progression at 6 months)

Clinical characteristics		Total (n: 475)	No ST in -30D (n: 393)	ST in -30D (n: 82)	No ST in D1-90 (n: 320)	ST in D1-90 (n: 155)	No ST > 6 m (n: 103)	ST > 6 m (n: 79)
Sex	Male	317 (66.7%)	262 (66.7%)	55 (67.1%)	218 (68.1%)	99 (63.9%)	73 (70.9%)	54 (68.3%)
	Female	158 (33.3%)	121 (33.3%)	27 (32.9%)	102 (31.9%)	56 (36.1%)	30 (29.1%)	25 (31.7%)
Type of tumor	NSCLC	161 (33.9%)	125 (31.8%)	36 (43.9%)	98 (30.6%)	63 (40.6%)	36 (35.0%)	33 (41.8%)
	Urothelial	82 (17.2%)	71 (18.1%)	11 (13.4%)	65 (20.3%)	17 (11.0%)	11 (10.7%)	10 (12.7%)
	Renal	65 (13.7%)	58 (14.8%)	7 (8.5%)	45 (14.1%)	20 (12.9%)	15 (14.5%)	15 (19.0%)
	Melanoma	53 (11.2%)	39 (9.9%)	14 (17.1%)	28 (8.8%)	25 (16.1%)	9 (8.8%)	6 (7.6%)
	Head/neck	52 (10.9%)	45 (11.5%)	7 (8.6%)	37 (11.6%)	15 (9.7%)	15 (14.6%)	9 (11.4%)
	GI	15 (3.2%)	13 (3.3%)	2 (2.4%)	10 (3.1%)	5 (3.2%)	5 (4.8%)	1 (1.2%)
	Others	47 (9.9%)	42 (10.6%)	5 (6.1%)	37 (11.5%)	10 (6.5%)	12 (11.6%)	5 (6.3%)
Type of ICI	Anti-PD-1	306 (64.4%)	259 (65.9%)	47 (57.3%)	213 (66.6%)	93 (60.0%)	73 (70.9%)	58 (73.4%)
	Anti-PD-L1	97 (20.4%)	82 (20.9%)	15 (18.3%)	71 (22.2%)	26 (16.8%)	13 (12.6%)	12 (15.2%)
	Anti-PD-1 + anti-CTLA-4	63 (13.3%)	44 (11.2%)	19 (23.2%)	30 (9.4%)	33 (21.3%)	16 (15.5%)	9 (11.4%)
	Anti-CTLA-4	9 (1.9%)	8 (2.0%)	1 (1.2%)	6 (1.8%)	3 (1.9%)	1 (1.0%)	0 (0%)
Line of treatment	1st line	200 (42.1%)	170 (43.3%)	30 (36.6%)	137 (42.8%)	63 (40.1%)	58 (56.3%)	37 (46.9%)
	2nd line	205 (43.2%)	166 (42.2%)	39 (47.6%)	139 (43.4%)	66 (42.6%)	33 (32.0%)	34 (43.0%)
	3rd line or further	70 (14.7%)	57 (14.5%)	13 (15.8%)	44 (13.8%)	26 (17.3%)	12 (11.7%)	8 (10.1%)
ECOG PS prior to ICI	0	147 (31.0%)	131 (33.3%)	16 (19.8%)	112 (35.0%)	35 (22.7%)	47 (45.6%)	31 (39.2%)
	1	254 (53.6%)	215 (54.7%)	39 (48.1%)	169 (52.8%)	85 (55.2%)	47 (45.6%)	45 (47.0%)
	2	66 (13.9%)	43 (11.0%)	23 (28.4%)	36 (11.3%)	30 (19.5%)	8 (7.8%)	3 (3.8%)
	3 or 4	7 (1.5%)	4 (1.0%)	3 (3.7%)	3 (0.9%)	4 (2.6%)	1 (1.0%)	0 (0%)
Disease burden prior to ICI	Metastases ≥ 3 organs	156 (33.5%)	111 (28.8%)	45 (55.6%)	90 (28.9%)	66 (42.6%)	26 (26.3%)	19 (24.7%)
	Brain metastases	74 (15.6%)	38 (9.7%)	36 (43.9%)	28 (8.8%)	46 (29.7%)	11 (10.8%)	14 (17.7%)
	Liver metastases	120 (25.3%)	98 (25.0%)	22 (26.8%)	78 (24.5%)	42 (27.1%)	19 (18.6%)	15 (19.0%)
	Lung metastases	216 (45.6%)	173 (44.1%)	43 (52.4%)	139 (43.6%)	77 (49.7%)	44 (43.1%)	32 (40.5%)
	Bone metastases	133 (28.1%)	101 (25.8%)	32 (39.0%)	78 (24.5%)	55 (35.5%)	23 (22.6%)	15 (19.0%)

### Association between steroids and clinical outcomes

**Fig. 1:**
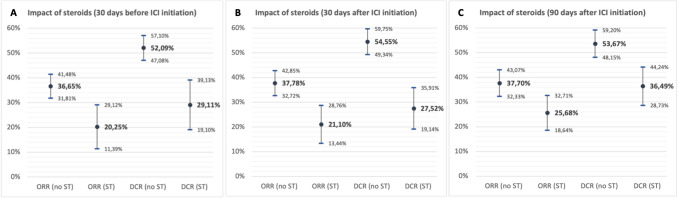
Association of steroids exposure with ICI clinical outcomes. A significant decrease in the objective response rate (ORR) and disease control rate (DCR) was observed in patients exposed steroids within the 30 days before (**A)**, 30 days after (**B)** and 90 days after ICI initiation (**C)**. For each value, horizontal bars represent the upper and lower limits of 95% confidence intervals (CI), estimated by two-sample test of proportions

In our cohort, exposure to steroids around ICI initiation was correlated with remarkably worse clinical outcomes. Compared to steroid-naïve patients, those exposed to steroids within 30 days before C1 had significantly lower ORR (20.3% vs. 36.7%; *p* < 0.01) and DCR (29.1% vs. 52.1%; *p* < 0.001) *(*Fig. 1A*)*.

Similar results were observed for the use of steroids within 30 days after C1, with a significant decrease in ORR (21.1% vs. 37.78%; *p* < 0.01) and DCR (27.5% vs. 54.6%; *p* < 0.001) *(*Fig. 1B*)*, and 90 days after C1, with a significantly lower ORR (25.7% vs. 37.7%; *p* = 0.011) and DCR (36.5% vs. 53.7%; *p* < 0.01) *(*Fig. 1C*)*. Among patients without evidence of progression at 6 months, those exposed to steroids (> 6 m) did not seem to have worse outcomes, and there was a non-significant trend for longer PFS when compared to patients without late steroid exposure (median PFS 23.2 vs. 17.6 months; hazard ratio [HR]: 0.68; 95% confidence interval [CI] 0.46–1.02).

### Impact of steroids dosage

**Fig. 2:**
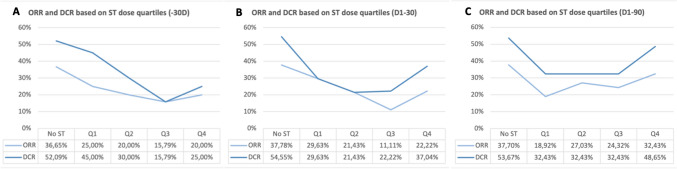
Correlation of steroid cumulative doses (CD) with clinical outcomes. A negative correlation between DCR and CD was found both within 30 days before (**A)** and 30 days after C1 (**B**), but no statistical differences were observed when considering CD within 90 days after C1 (**C**)

When patients were divided into quartiles (Q) based on the total steroid dose received within 30 days before C1 (-30D), an inverse correlation between the cumulative dosage and clinical benefit was observed. The dose upper limit for each group was 2.36 mg/kg/30d (Q1), 4.05 mg/kg/30d (Q2), 7.94 mg/kg/30d (Q3) and 40.73 mg/kg/30d (Q4). The ORR for each group was: 36.7% (no steroids), 25% (Q1), 20% (Q2), 15.8% (Q3), and 20% (Q4) (*p* = 0.093); the DCR for each group was: 52.1% (no steroids), 45% (Q1), 30% (Q2), 15.8% (Q3) and 25% (Q4) (*p* = 0.001) *(*Fig. 2A*)*. The mean cumulative dose of steroids was significantly higher in non-responders than in responders to ICI (1.59 mg/kg/30d [95% CI 1.02–2.16] vs. 0.58 mg/kg/30d [95% CI 0.22–0.94]; *p* = 0.0175).

Similar results were obtained for the cumulative dose within 30 days after C1 (D1-30) (*Fig. 2B*). The dose upper limit for each group was 2.9 mg/kg/30d (Q1), 6.15 mg/kg/30d (Q2), 11.41 mg/kg/30d (Q3), and 48.08 mg/kg/30d (Q4). The ORR for each group was 37.8% (no steroids), 29.6% (Q1), 21.4% (Q2), 11.1% (Q3) and 22.2% (Q4) (*p* = 0.012). The DCR for each group was 54.6% (no steroids), 29.6% (Q1), 21.4% (Q2), 22.2% (Q3), and 37.0% (Q4) (*p* < 0.001) *(*Fig. 2B*)*. The mean cumulative dose was also higher in non-responders to ICI (2.48 mg/kg/30d [95% CI 1.81–3.14] vs. 1.23 mg/kg/30d [95% CI 0.44–2.01] in responders; *p* = 0.024).

No clear correlation was found between the cumulative steroid dose within 90 days after C1 (D1-90) and clinical outcomes *(*Fig. 2C*)*, and the difference between the mean cumulative doses for responders and non-responders was not statistically significant (*p* = 0.33). When the impact of steroid dosage after 6 months from C1 on survival outcomes was analyzed, the correlation seemed to reverse, with significantly higher PFS for those patients receiving higher doses: 17.6 months (no ST), 17.3 months (Q1, dose upper limit 6 mg/kg/30d), 37.1 months (Q2, upper limit 19.2 mg/kg/30d), 19.9 months (Q3, upper limit 48.9 mg/kg/30d), and 42.1 months (Q4, upper limit 145.4 mg/kg/30d) (HR: 0.86, 95% CI 0.75–0.99; *p* = 0.039).

### Association between irAEs and clinical outcomes

The presence of immune-related toxicity was correlated with a higher ORR (46.6% vs. 27.8%; *p* < 0.001), longer PFS (11.6 vs. 3.6 months; HR: 0.59; 95% CI 0.47–0.75) and longer OS (20.7 vs 8.0 months; HR: 0.56; 95% CI 0.45–0.69). The association between irAEs and survival outcomes was confirmed by multivariable analysis including PS, tumor type, disease burden, and line of treatment (*p* < 0.001).

### Impact of the neutrophil-to-lymphocyte ratio

In our cohort, the baseline neutrophil-to-lymphocyte (N/L) ratio was positively correlated with irAEs and negatively correlated with clinical outcomes. A N/L ratio > 4 was led to a significantly lower DCR (39.6% vs. 54.6%; *p* = 0.001) and incidence of irAEs (26.4% vs. 39.5%; *p* < 0.01). Interestingly, the proportion of patients with a baseline N/L ratio > 4 was significantly higher among those exposed to steroids within 30 days before ICI initiation (67.1% vs. 37.7%; *p* < 0.0001).

## Discussion

The results of our study in a multi-tumor cohort suggest a negative effect of steroids use on the efficacy of ICI, particularly when the exposure occurs around treatment initiation, and seemingly with a dose-dependent pattern. Interestingly, baseline exposure to steroids seems particularly harmful and has been correlated with N/L ratio.

### The relevance of timing

Our results are consistent with those of previous studies, suggesting a potential negative effect of early steroid exposure around ICI initiation. A meta-analysis of 15 retrospective studies, including patients with brain metastases (*n*: 1102), showed significantly worse PFS (HR: 2.00, 95% CI 1.37–2.91) and OS (HR: 1.84, 95% CI 1.22–2.77) for those exposed to steroids [16]. In a retrospective study of 151 patients with metastatic NSCLC, the early use of steroids (within 28 days after ICI initiation) was correlated with worse DCR (odds ratio [OR]: 0.32; 95% CI 0.14–0.71), PFS (HR: 1.8; 95% CI 1.20–2.80) and OS (HR: 2.6; 95% CI 1.70–4.10) [17].

In our cohort, the negative association of steroids with clinical responses also seemed to prevail around ICI initiation, with a significant decrease in the ORR and DCR among patients exposed between -30D and D90 from C1. However, this effect was not observed for steroid exposure after 6 months in long-term responders, with a tendency for longer PFS when compared to the steroid-naïve group. It should be noted that these patients must at least have survived until starting steroids, thus immortal-time bias cannot be discarded.

In addition, since early exposure to steroids (-30D to D90) is mainly due to cancer-related conditions, some of which (as symptomatic brain metastases) inherently imply poor prognosis, and late exposure in responding patients (> 6 m) is mostly due to irAEs, which are in contrast correlated with better outcomes, there might be a potential indication bias in the observed time-dependent effect of steroid exposure.

Although the association between irAEs and favorable outcomes may be prone to immortal-time bias (patients must at least have lived until the onset of irAEs), there is solid evidence in the literature regarding the positive prognostic impact of immune toxicity. In a meta-analysis of 51 studies, the development of irAEs was associated with better survival outcomes among patients with metastatic melanoma (OS HR: 0.46, 95% CI 0.35–0.62; PFS HR: 0.51, 95% CI 0.42–0.63) and advanced NSCLC (OS HR: 0.40, 95% CI 0.30–0.51; PFS HR: 0.46, 95% CI 0.39–0.54) [18]. Similar results were obtained by Zhou et al. [19] in a meta-analysis of 30 studies including 4971 patients, with a significant benefit in OS (HR: 0.54; 95% CI 0.45–0.65) and PFS (HR: 0.52; 95% CI 0.44–0.61) for those who developed irAEs, particularly low-grade endocrine and cutaneous reactions.

In addition, some studies have suggested a negative impact of steroids only when indicated for cancer-related events, but not for the management of irAEs. A systematic review and meta-analysis of 16 studies (*n* = 4045) showed an increased risk of death and progression among patients treated with ICI receiving steroids for supportive care (HR: 2.5; 95% CI 1.41–4.43) or brain metastases (HR: 1.51; 95% CI 1.22–1.87), but not among those with irAEs [20]. Similar results have been obtained in other studies that analyzed large cohorts of patients treated with ICI for advanced NSCLC [21, 22].

However, Maslov et al. [23] evaluated the outcomes of 247 patients treated with ICI and concurrently exposed to steroids, analyzing the effect of steroid timing, and reported significantly longer PFS for those exposed to steroids within the first 2 months after ICI initiation, irrespective of the clinical indication. The median PFS was significantly longer when steroids were prescribed after 2 months from C1, both in the group with irAEs (HR 0.33; *p* < 0.0001) and in the group treated with steroids for other reasons (HR 0.27; *p* < 0.0001).

Although a potential skew related to steroid indication cannot be completely dismissed, these results suggest that steroid biological effects might be intrinsically time-dependent, probably more relevant at impairing the achievement of a successful T cell antitumor response than at inducing loss of clinical benefit in responders, thus prevailing around ICI initiation, irrespective of indication.

### The relevance of dosage

Although the impact of steroid use on ICI outcomes has been assessed in several studies, the influence of dosage has not been previously analyzed in detail. In our cohort, an inverse correlation was found between cumulative doses and clinical outcomes, with higher doses leading to lower ORR and DCR, both within 30 days before and 30 days after C1. These results should be interpreted with caution, as the sample size for each quartile is probably low considering the heterogeneity of patients. The finding of significantly higher doses around ICI initiation among non-responders supports the hypothesis of a dose-dependent effect.

Interestingly, even low doses of steroids seemed to lead to worse DCR compared with no steroid exposure. Most ICI clinical trials have excluded patients with baseline daily doses ≥ 10 mg prednisone, usually considering doses below that threshold as physiological. However, in our cohort, patients from the first quartile of baseline steroid dose (< 2.36 mg/kg/30d, nearly half the cumulative dose of a 70 kg patient receiving 10 mg prednisone per day) had significantly worse DCR than steroid-naïve patients, questioning the assumption that there is a ‘safe’ dose of steroids prior to initiation of immunotherapy.

After the first month of treatment, this association seemed to attenuate (no significant differences were observed when steroids in D1-90 were analyzed together) and eventually became inverted, since higher doses after 6 months from C1 were significantly associated with longer PFS. Again, the better outcomes of patients with late exposure to high-dose steroids could be explained by the higher incidence of irAEs in this subgroup of long-term responders, provided the established correlation between irAEs and clinical benefit.

Considering that some patients may have received higher peak doses a few times, while others may have been exposed to lower doses for longer periods, data obtained from cumulative dosage are difficult to extrapolate to clinical practice. Although this poses a limitation to our analysis, these results support the central hypothesis of a dose-dependent detrimental effect.

### The relevance of immune cells profile

The neutrophil-to-lymphocyte (N/L) ratio seems to predict clinical response and immune-related toxicity in patients treated with ICI. In a retrospective study of 1714 patients with 16 different types of solid tumors, Valero et al. [24] found a significant association between a higher N/L ratio and poorer PFS and response rates. Combining the N/L ratio and TMB, the benefit of ICI was significantly higher in the N/L-low/TMB-high group than in the N/L-high/TMB-low group (OR: 3.22; 95% CI 2.26–4.58). Xie et al. [25] published a meta-analysis of 14 studies incorporating 1751 participants, showing that elevated pre-treatment N/L ratio was associated with poorer OS (HR: 2.61; 95% CI 1.77–3.86) and PFS (HR: 1.74; 95% CI 1.34–2.27). In a meta-analysis of 7 published articles on the utility of the baseline N/L ratio, Sacdalan et al. [26] found worse OS (HR: 1.92; 95% CI 1.29–1.87) and PFS (HR: 1.66; 95% CI 1.38–2.01) among patients with higher N/L ratios across several tumor types. Similar results were obtained by Takenaka et al. [27] in a meta-analysis of 14 studies with 929 patients with head and neck tumors, as well as individual studies analyzing large cohorts of patients with advanced NSCLC [28–30], renal cell carcinoma [31, 32], pancreatic cancer [33] and upper gastro-intestinal cancer [34, 35], among others.

Exposure to steroids appears to modulate the N/L ratio during ICI treatment. Fucà et al. [17] found a correlation between steroids use and a higher median N/L ratio, both 4 and 6 weeks after C1, hypothesizing that steroids may hinder antitumor response by modulating peripheral blood immune cells. The effect of steroids may be particularly detrimental in patients with a lower N/L ratio. Lauko et al. [36] studied 171 patients with brain metastases from NSCLC, reporting decreased OS (10.5 vs. 17.9 months; *p* = 0.03) and intracranial PFS (5.0 vs. 8.7 months; *p* = 0.045) in those with upfront steroids; interestingly, OS differences were only significant in the subgroup of patients with a baseline N/L ratio < 4, and there was a strong interaction between the N/L ratio and upfront steroids when modeled together (*p* = 0.0008). Our study is the first to demonstrate a correlation between steroid use before ICI initiation and a higher baseline N/L ratio.

A reasonable potential critique of this study is the reflection that the baseline steroid requirement could be driven by intrinsically tumor-related bad-prognosis scenarios, such as symptomatic brain metastases, with steroid use being the consequence and not the cause of worse clinical results. Nevertheless, since the N/L ratio has thoroughly been proven to be associated with poor ICI outcomes, the currently observed correlation between steroids and higher N/L ratios, suggests that there is indeed a biological basis supporting the hypothesis of steroid-induced impairment of ICI efficacy.

## Conclusions

Our study supports the hypothesis of a negative effect of early exposure to steroids on the outcomes of immune checkpoint inhibitors because of their diverse immunosuppressive properties, leading to a significant decrease in the response and disease control rates. Their detrimental effect seemed to prevail around ICI initiation (at least from -30D to D90) and was not observed in our cohort after 6 months of treatment. This finding might be biased by the positive prognostic impact driven by immune-related toxicity, which mostly explains the late exposure to steroids in long-term responders, as opposed to cancer-related complications leading to their early use around ICI initiation, although previous clinical evidence has suggested an intrinsically time-dependent biological effect.

Although the influence of steroid dose has not been previously analyzed, our study suggests an inverse correlation between DCR and cumulative dose around ICI initiation. The finding of significantly higher cumulative doses among non-responders supports the dose-dependent effect hypothesis. In addition, the remarkable drop in the DCR observed in the lowest-dose quartile of patients from our cohort suggests that even the usually considered as ‘physiological’ low doses of steroids might interfere with ICI efficacy.

Interestingly, steroid use within 30 days before ICI initiation correlated with higher baseline neutrophil-to-lymphocyte ratios, which led to significantly poorer clinical outcomes. As an established biomarker for response to ICI, the demonstration that the N/L ratio is modulated by steroids challenges the idea of steroid exposure as a mere bystander in the natural history of worse-prognosis tumors, providing a clue to understanding the biological basis of their detrimental influence on ICI outcomes. Further research and prospective validation of these results would be of great value to better understand the effects of steroids and optimize their use in patients undergoing treatment with immunotherapy.

## Data Availability

The original data that support the findings of this study are available upon request.

## Abbreviations

APC: Antigen presenting cells
> 6 m: After 6 months from C1
C1: First cycle
CD: Cumulative doses
CEIC: Clinical Investigation Ethical Committee
CI: Confidence interval
CTLA-4: Cytotoxic T lymphocyte associated protein 4
D1-30: 30 Days after C1
D1-90: 90 Days after C1
− 30D: 30 Days before C1
DCR: Disease control rate
ECOG: Eastern Cooperative Oncology Group
GR: Glucocorticoid receptor
HIV: Human immunodeficiency virus
HR: Hazard ratio
ICI: Immune checkpoint inhibitors
irAEs: Immune-related adverse events
LAG-3: Lymphocyte activation gene 3
MDSCs: Myeloid-derived suppressor cells
MHC: Major histocompatibility complex
NK: Natural killer
N/L ratio: Neutrophil-to-lymphocyte ratio
NSCLC: Non-small cell lung cancer
ORR: Objective response rate
OS: Overall survival
PD-1: Programmed cell death protein 1
PD-L1: Programmed cell death protein ligand 1
PFS: Progression-free survival
PS: Performance status
Q: Quartile
RWD: Real-world data
ST: Steroids
TAA: Tumor-associated antigens
TAMs: Tumor-associated macrophages
TCR: T cell receptor
TGF-β: Tumor growth factor β
TIM-3: T cell immunoglobulin domain and mucin domain 3
TILs: Tumor-infiltrating lymphocytes
TME: Tumor microenvironment
TMB: Tumor mutational burden
T-reg: Regulatory T cells
